# Regional mapping of CSF1R-positive microglia in neurodegenerative diseases and progressive MS, with exploratory presynaptic marker analyses

**DOI:** 10.1186/s12974-026-03945-6

**Published:** 2026-07-04

**Authors:** Mahsa S. Bavarsad, Felipe L. Pereira, Marina M. Reinhardt, Abhijit Satpati, Salvatore Spina, William W. Seeley, William Jagust, Gil D. Rabinovici, Ari Green, Lea T. Grinberg

**Affiliations:** 1 Memory and Aging Center, Weill Institute for Neurosciences, University of California San Francisco, San Francisco, CA USA; 2 Departments of Laboratory Medicine and Pathology and Neurosciences, Mayo Clinic Florida, 4500 San Pablo Rd, Jacksonville, FL 32250 USA; 3 Department of Quantitative Health Sciences, Mayo Clinic, Jacksonville, FL USA; 4 Helen Wills Neuroscience Institute, University of California, Berkeley, CA USA; 5 Department of Neurology, University of California San Francisco, San Francisco, CA USA

**Keywords:** Microglia, CSF1R, Synapse/synaptic, Alzheimer’s Disease, Frontotemporal Dementia, Multiple Sclerosis

## Abstract

**Background:**

Microglial colony-stimulating factor-1 receptor (CSF1R) is a therapeutic and imaging target, yet the regional, disease-specific distribution of CSF1R-positive microglia in the human brain remains incompletely defined, limiting interpretation of emerging CSF1R-PET signals. We sought to build a cross-disease, multi-region, quantitative map of CSF1R-positive microglia in neurodegenerative conditions and progressive multiple sclerosis (MS) lesions, with an exploratory comparison to presynaptic marker burden.

**Methods:**

CSF1R mRNA‑positive microglia were quantified by RNAscope across six cortical regions (MFG, IFG, ITG, AG, CA1, EC) in early‑onset Alzheimer's disease (EOAD), late‑onset AD (LOAD), progressive supranuclear palsy (PSP), and frontotemporal lobar degeneration with TDP-43 inclusions due to progranulin mutation (FTLD‑GRN), and in primary and secondary progressive MS (PPMS, SPMS) within cortical gray‑matter plaques, plaque-adjacent gray matter and white matter. Positivity was defined a priori as ≥ 3 puncta with housekeeping‑probe pass and negative‑control verification, counting blinded, and densities were cortical‑thickness corrected. Iba-1 immunolabeling verified microglial identity. Western blot provided protein‑level verification. We explored ROI‑level associations of CSF1R with SV2A and synaptophysin previously measured in the same regions/cases.

**Results:**

In neurodegeneration, increases were smaller and region‑specific (e.g., EOAD—ITG/CA1; LOAD—AG; PSP—AG; FTLD‑GRN—IFG/ITG/AG/EC), with minimal white‑matter change. In progressive MS, gray-matter CSF1R-positive microglia densities did not differ from controls, whereas SPMS white matter was increased. Exploratory analysis showed that CSF1R and SV2A were positively associated across ROIs in neurodegenerative diseases (e.g., PSP approximately ρ = 0.66), and weakest in LOAD; synaptophysin showed similar patterns, suggesting that regions with higher CSF1R-positive microglia density can coincide with relative preservation of presynaptic markers.

**Conclusions:**

A cross‑disease, region‑resolved map reveals region‑specific changes in CSF1R + cell density in neurodegeneration, but only white matter in MS. These findings provide the histological context needed to interpret future CSF1R‑PET. Prospective studies pairing CSF1R‑PET with SV2A‑PET and multiplex tissue profiling are warranted to define microglial states and synaptic outcomes in vivo.

**Supplementary Information:**

The online version contains supplementary material available at https://doi.org/10.1186/s12974-026-03945-6.

## Introduction

Neuroinflammation driven by microglia is a central feature of neurodegenerative diseases and multiple sclerosis (MS) [1, 2]. The colony-stimulating factor-1 receptor (CSF1R), expressed primarily by microglia, regulates their survival, proliferation, and function through CSF1 and IL-34 [3, 4], and is increasingly targeted to monitor and modulate microglial states in human disease. Elevated CSF1R expression has been observed in neurological diseases such as AD and MS, making it a potential diagnostic tool and therapeutic target [5–7].

Recent advances in molecular imaging have transformed the diagnosis of neurological conditions via the development of positron emission tomography (PET) tracers. Initial tracers targeting neuroinflammation, such as translocator protein (TSPO), demonstrated the unlocked potential of detecting neuroinflammation in living individuals. Despite some limitations of TSPO tracers, such as a lack of target specificity, poor signal-to-noise ratios, and genetically driven binding variability [8], the opportunity of detecting neuroinflammation in vivo has prompted the development of targeted alternatives, including ^11^C-CPPC, a CSF1R-specific radiotracer. Yet the regional and disease-specific distribution of CSF1R-positive microglia in the human brain remains incompletely defined, limiting interpretation of emerging CSF1R-PET signals [9, 10].

A cross-disease, region-resolved map of CSF1R-positive microglia across diseases—including progressive MS with lesion-level sampling—would provide the histological context needed to interpret CSF1R-PET across disorders and to understand when large dynamic ranges should be expected. We quantitatively mapped CSF1R mRNA-positive microglia by RNAscope across six cortical regions in early-onset Alzheimer’s disease (EOAD), late-onset Alzheimer’s disease (LOAD), progressive supranuclear palsy (PSP), frontotemporal lobar degeneration with TDP-43 inclusions due to GRN mutations (FTLD-GRN), and within cortical plaques and white matter in primary progressive MS (PPMS) and secondary progressive MS (SPMS), with supportive CSF1R protein analysis by Western blot. Together, this cross-disease, region-resolved map of CSF1R-positive microglia aims to provide the histological context needed to interpret CSF1R-PET across disorders and to guide the selection of brain regions and disease settings where CSF1R-based measurements are likely to be most informative.

Moreover, because microglia can support synapse maintenance in some contexts but contribute to synaptic loss in others, and preclinical manipulations of CSF1R signaling (including inhibition or microglial depletion) have produced mixed effects on synapses [10–14], we additionally conducted an exploratory to evaluate whether CSF1R microglia burden tracks with loss or relative preservation of presynaptic markers (synaptic vesicle protein 2 A [SV2A] and synaptophysin) previously mapped in the same regions and largely the same cases [15].

## Materials and methods

### Case characteristics and inclusion criteria

All post-mortem human brain tissue was obtained under IRB-approved protocols. Written informed consent was obtained from next of kin, and all procedures complied with the Declaration of Helsinki. Postmortem human brain tissue was sourced from the UCSF Neurodegenerative Disease Brain Bank (NDBB). The NDBB primarily acquires brain donations from patients evaluated at the UCSF Memory and Aging Center. In addition, post-mortem human brain tissues from individuals with MS were obtained from the Netherlands Brain Bank (NBB).

Neuropathological assessments were conducted using standardized protocols in alignment with internationally recognized criteria for neurodegenerative diseases [16–19]. The study design incorporated brain regions with varying degrees of vulnerability to the conditions under investigation, enabling a more comprehensive analysis and facilitating improved cross-disease comparisons. This study included 36 cases, encompassing healthy controls, EOAD, LOAD, PSP, FTLD-GRN and MS (Table 1).

**Table 1:** Demographic, neuropathological, and clinical characteristics of cases included in the study

A. Neurodegenerative cohort used for RNAscope in situ hybridization and Western blotting									
**Case**	**Pathological diagnosis**	**Sex**	**Age at onset (yr)**	**Age at death (yr)**	**PMI (hr)**	**Braak NFT stage**	**Fixation duration (hr)**	**Western blotting performed**	**Histological quantification**
GL27	Control	M	NA	65	11.4	II	Long fix	—	+
GL17	Control	F	NA	67	19.4	0	72	+	+
GL3	Control	M	NA	76	8.2	II	72	+	—
GL15	Control	F	NA	82	9.5	I	72	+	+
GL2	Control	F	NA	86	7.8	II	72	+	+
GL24	EOAD	F	44	58	13.1	VI	72	+	—
GL18	EOAD	F	53	60	13.0	VI	72	+	+
GL25	EOAD	M	50	62	6.0	VI	Long fix	+	—
GL23	EOAD	F	51	62	8.6	VI	72	—	+
GL13	EOAD	M	49	63	6.8	VI	72	+	+
GL9	EOAD	F	54	64	7.3	VI	72	+	+
GL1	EOAD	M	58	70	9.0	VI	72	+	+
GL22	LOAD	M	61	69	11.5	VI	72	—	+
GL16	LOAD	M	65	71	9.2	VI	72	+	+
GL6	LOAD	F	67	73	12.5	VI	72	+	+
GL8	LOAD	F	69	75	6.8	VI	72	+	+
GL26	LOAD	M	79	85	11.1	V	Long fix	+	—
GL4	LOAD	M	74	86	10.8	VI	72	+	+
GL10	LOAD	F	77	87	6.1	VI	72	+	+
GL19	FTLD-GRN	F	56	64	10.5	I	72	—	+
GL7	FTLD-GRN	M	59	64	7.2	II	72	—	+
GL12	FTLD-GRN	M	62	72	7.2	VI	72	—	+
GL20	FTLD-GRN	F	65	73	7.4	I	72	—	+
GL11	PSP	F	54	70	24.8	I	72	—	+
GL21	PSP	M	60	68	13.8	I	72	—	+
GL5	PSP	F	62	69	18.5	II	72	—	+
GL14	PSP	M	71	80	7.9	II	72	—	+

B. Multiple sclerosis cohort used for RNAscope in situ hybridization									
**Subject**	**Diagnosis**	**Sex**	**Age at death (yr)**	**Disease duration (yr)**	**PMI (hr)**	**Fixation duration (hr)**	**Total GM plaques per tissue block**	**Western blotting performed**	**Histological quantification**
GL28	PPMS	F	54	31	9.3	Long fix	3	—	+
GL29	PPMS	F	51	35	9.8	Long fix	1	—	+
GL30	PPMS	M	59	12	10.8	Long fix	2	—	+
GL31	PPMS	M	56	33	9.6	Long fix	7	—	+
GL32	SPMS	F	60	7	10.7	Long fix	3	—	+
GL33	SPMS	F	47	29	8.6	Long fix	5	—	+
GL34	SPMS	F	48	22	11.8	Long fix	1	—	+
GL35	SPMS	M	65	36	9.5	Long fix	2	—	+
GL36	SPMS	M	70	38	9.4	Long fix	1	—	+

NA indicates not available or not applicable; — indicates not performed. MS tissue block dimensions (depth × length × width) were approximately 0.5 cm × (1–2 cm) × (1–2 cm). Control cases (GL15, GL17, GL2, GL27) are listed in Panel A and served as controls for MS analyses

*Abbreviations yr* year, *hr* hour, *NFT* Neurofibrillary tangle, *PMI* Post-mortem interval, *EOAD* Early-onset Alzheimer’s disease, *LOAD* Late-onset Alzheimer’s disease, *FTLD-GRN* FTLD-TDP type A due to progranulin mutation, *PSP* Progressive supranuclear palsy, *GM* Gray matter, *PPMS* Primary progressive multiple sclerosis, *SPMS* Secondary progressive multiple sclerosis

Cases were selected based on strict inclusion criteria. Healthy controls were defined as individuals with no neuropathological diagnoses and confirmed as cognitively normal at the time of death. Neurodegenerative disease cases had to meet neuropathological diagnostic standards for their respective conditions and exhibit no coexisting neuropathological diagnoses that could potentially influence CSF1R dysregulation. All EOAD, LOAD, and PSP cases were sporadic, with PSP cases presenting as Richardson’s syndrome. EOAD cases were characterized by symptom onset before the age of 65. All FTLD-GRN cases carried a pathogenic GRN variant and demonstrated FTLD-TDP type A pathology at autopsy.

Inclusion criteria for neurodegenerative cases and healthy controls required the availability of tissue samples from six predefined regions of interest: the middle frontal gyrus (MFG), inferior frontal gyrus (IFG), inferior temporal gyrus (ITG), Cornu Ammonis sector 1 of the hippocampus (CA1), entorhinal cortex (EC), and angular gyrus (AG). For MS cases, post-mortem blocks containing both neocortex and subcortical white matter were selected from MRI and histological pictures provided by the NBB (Fig. S1). We chose all available blocks containing gray matter plaques. Cases included primary progressive multiple sclerosis (PPMS) cases and secondary progressive multiple sclerosis (SPMS) cases. MFG of healthy control tissue was used as controls for the MS cases.

To ensure assay validity we only included cases with high tissue integrity defined by RNAscope assays (Advanced Cell Diagnostics [ACD]). Inclusion criteria required housekeeping genes (PPIB, UBC, or POLR2A) to score at least 2 (≥ 4 puncta per cell). Negative RNAscope control probes confirmed low background staining,).

### Labelling of CSF1R-positive cells based on in situ hybridization (RNAscope)

Because currently available anti-CSF1R antibodies did not yield robust, specific immunolabeling in FFPE human cortex despite extensive optimization and consultation with other laboratories, RNAscope was selected as the primary staining assay because it provides sensitive, single cell, spatially-resolved detection of CSF1R transcripts making it suitable for quantitative mapping.

**Fig. 1:**
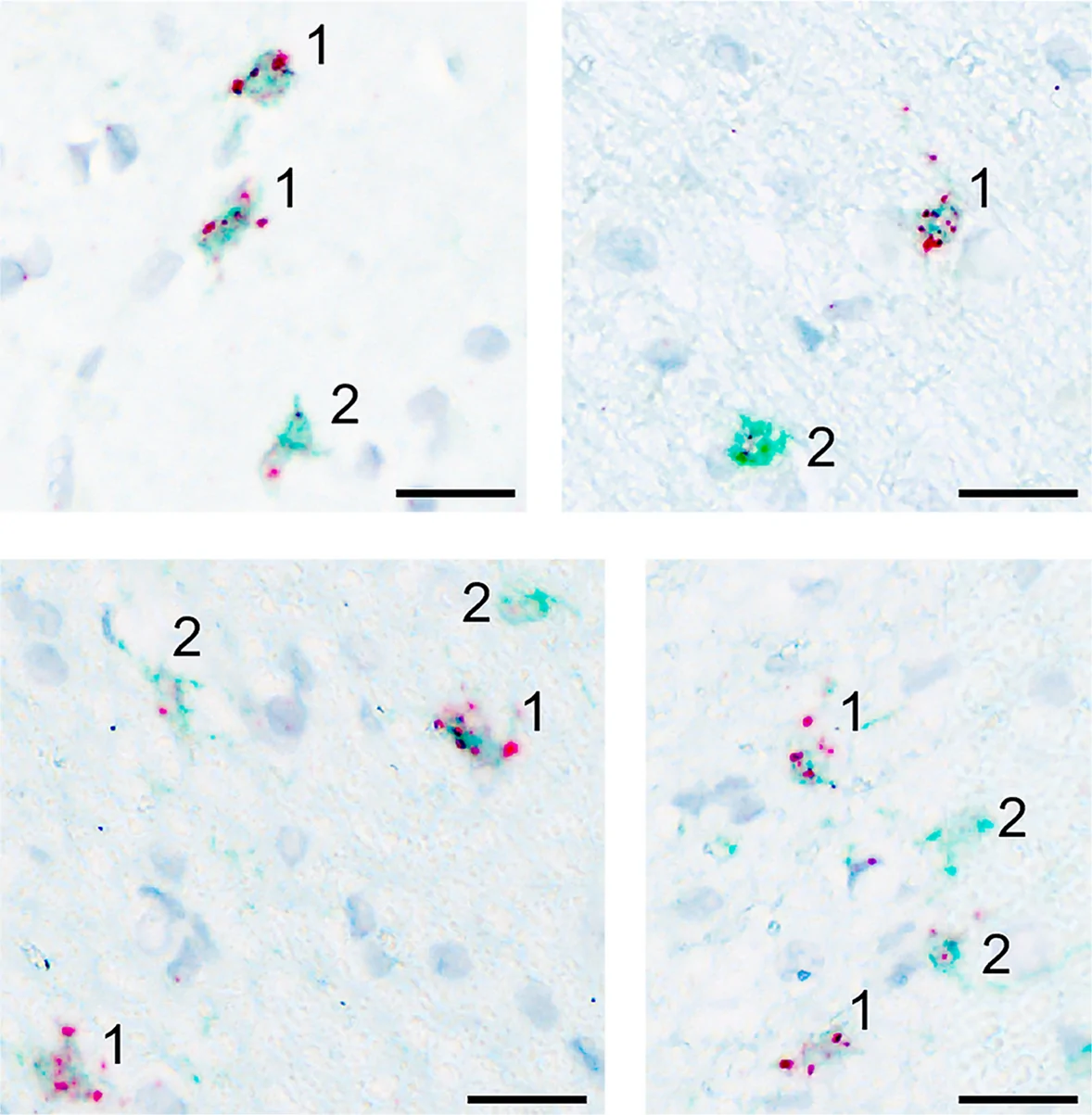
CSF1R transcripts colocalize with Iba-1-positive microglia within the gray matter (GM) of the human inferior temporal gyrus (ITG) in a healthy control case (top row) and an Alzheimer's disease (AD) case (bottom row).. Formalin-fixed paraffin-embedded (FFPE) brain sections were dual-labeled using immunohistochemistry (IHC) for Iba-1 protein (green; microglial marker) and in situ hybridization (ISH) for CSF1R mRNA (red puncta). CSF1R transcripts colocalize with Iba-1-immunoreactive microglia, confirming CSF1R expression in microglial cells. Numbered labels indicate examples of Iba-1 positive microglia that meet the CSF1R positivity threshold (≥ 3 CSF1R puncta; 1) and Iba-1 positive microglia that do not meet this threshold (< 3 CSF1R puncta; 2). Scale bars: 10 μm

Most human brain tissue from healthy controls and disease groups, including EOAD, LOAD, PSP, and FTLD-GRN, was fixed in 10% buffered formalin for 72 h prior to paraffin embedding, whereas one control, two ND and all MS brain tissues were fixed in 4% formaldehyde solution for approximately four weeks before sampling and paraffin embedding due to differences in brain banks protocols. Formalin-fixed paraffin-embedded (FFPE) brain sections. (5 μm-thick) underwent in situ hybridization using the RNAscope 2.5 HD Assay-Red Detection Kit (ACD, Cat. No. 322360) to detect CSF1R mRNA (ACD, Cat. No. 518001), following the manufacturer's protocol. Briefly, tissue sections were deparaffinized, hybridized with a specific probe, subjected to signal amplification, and washed thoroughly before detection using the Fast Red substrate. Sections were then counterstained with hematoxylin (Cancer Diagnostics, Cat. No. SH5777) and mounted with EcoMount (Biocare Medical, Cat. No. EM897L). To validate assay performance, ACD’s Hs-UBC probe (Cat. No. 310041) was used as a positive control. CSF1R positivity manifests as red puncta (Fig. 1).

### Validation of cell-type specificity of CSF1R mRNA Detection by RNAscope

CSF1R is predominantly expressed by microglia, although low-level expression has been reported in a subset of neurons under homeostatic and pathological conditions [20]. As a precautionary measure prior to performing spatial mapping of CSF1R-positive cells, we conducted an experiment to assess the cell-type specificity of CSF1R mRNA detected by RNAscope. In short, we performed dual labeling using RNAscope for CSF1R mRNA in combination with immunohistochemistry for ionized calcium-binding adaptor molecule 1 (Iba-1), a microglial marker, in cortical tissue from a healthy control and an individual with AD. In both samples, CSF1R mRNA signal was localized almost exclusively to Iba-1-positive microglia, establishing cell identity for subsequent quantification (Fig. 1).

### Quantification of CSF1R-positive microglia density in neurodegenerative disease groups

Counting was performed blinded to diagnosis by a trained rater. For RNAscope image acquisition and quantification, slides were scanned with a calibrated Zeiss Axioscan at 20 × magnification and images were processed in ZEN (ZEISS ZEN 3.5, Blue Edition). Fast Red puncta indicating CSF1R mRNA were quantified in defined square counting frames (193,405.39 μm^2^) placed by a randomly positioned grid; 16 neocortical frames per slide were sampled (8 Gy matter, 8 white matter) and, for hippocampal formation slides, 4 frames each for EC and CA1. Because white matter within the hippocampal formation is limited, CA1/EC white-matter measurements were obtained from parahippocampal gyrus white matter sampled consistently across cases, excluding fields adjacent to the gray-white boundary. Fields with folds, tears, hemorrhage, or edge artifacts were excluded from counting a priori. A cell was considered RNAscope-positive for CSF1R if it contained ≥ 3 Fast Red puncta. The ≥ 3-puncta threshold follows the manufacturer’s recommended semi-quantitative scoring framework (Advanced Cell Diagnostics (ACD) RNAscope reference/scoring guide (ACD RNAscope Reference Guide) and aligns with recent studies applying single-cell RNAscope scoring in human brain tissue [21–24] (Fig. 1).

To assess scoring consistency, a randomly selected 10% subset of fields was independently re-counted by a second rater.

As the counting area serves as the denominator in the density equation, atrophy due to disease or edema following a peri-agonal event, among other factors can artificially affect cell burden. These distortions are analogous to partial volume effects in neuroimaging and similarly require correction. To minimize artifactual distortions in CSF1R-positive microglia density, we applied a correction based on expected cortical thickness, as we demonstrated previously [15]. First, we measured cortical thickness along the gyral wall of each slide, ensuring all measurements were taken perpendicular to the gray–white matter boundary. We then calculated the mean cortical thickness for healthy control cases in each brain region. For each disease slide, we computed a thickness ratio relative to mean cortical thickness of control cases for the respective brain area. Next, we corrected the raw CSF1R-positive microglia density values by multiplying them by these thickness ratios.

### Quantification of CSF1R-positive microglia density in multiple sclerosis

In MS cases, plaques were defined on digitized HLA-PLP-stained sections from slides adjacent/parallel to the CSF1R RNAscope sections (Fig. S1). Plaque borders were delineated on the Hla-PLP-stained images and transferred to the corresponding digitized CSF1R-stained sections. For each cortical gray-matter plaque, we also defined a plaque-adjacent penumbral region as the 500-um gray-matter zone surrounding the plaque border, and distant-appearing gray matter was sampled at least 3 mm from any plaque. Histologically unaffected white matter was analyzed separately. When multiple plaques were present in a case, each plaque and its penumbra were measured and analyzed independently while retaining case identity in the statistical model. Plaque rim and core were analyzed together. CSF1R-positive microglia densities were calculated by dividing the absolute number of positive cells by the measured ROI area converted to mm^2. For distant-appearing gray matter and unaffected white matter, five randomly selected counting frames per compartment were quantified per case; each frame measured 160,000 um^2^ (0.16 mm^2^). MFG values from the same control cases used for the neurodegenerative-disease analyses served as controls for the MS comparisons.

### Immunoblotting and quantitative analysis

Frozen brain samples of the IFG were obtained from six cases, including one EOAD, one LOAD, and four healthy controls (Table 1). Tissue (1 g) was homogenized using ice-cold RIPA buffer (RIPA Lysis and Extraction Buffer #89,900, Thermo Scientific™) supplemented with protease and phosphatase inhibitors (Pierce Protease and Phosphatase Inhibitor Mini Tablets #A32959, Thermo Scientific™). The homogenate was centrifuged at 21,000 g for 30 min at 4 °C, and the supernatant was collected and stored at −80 °C. Total protein concentration was determined using the Pierce™ Bradford Protein Assay Kit (#23,200, Thermo Scientific™) and a SpectraMax 384 Plus Microplate Reader (Molecular Devices).

For protein analysis, 25 µg of the extracted protein was diluted with LDS Sample Buffer (NuPAGE™ LDS Sample Buffer (4X) #NP0007, Invitrogen™) and reducing agents (NuPAGE™ Sample Reducing Agent (10X) #NP0009, Invitrogen™), then heated for 10 min at 77.7 °C. The samples were separated on 4–12% Bis–Tris gels (NuPAGE™ 4 to 12%, Bis–Tris, 1.0–1.5 mm, Mini Protein Gels, #NP0335BOX, Invitrogen™) using MOPS SDS Running Buffer (NuPAGE™ MOPS SDS Running Buffer (20X) #NP0001, Invitrogen™). Proteins were transferred to polyvinylidene difluoride (PVDF) membranes (Immun-Blot PVDF Membrane, 1,620,177, Bio-Rad), which were blocked with non-fat dry milk (Blotting-Grade Blocker, 1,706,404, Bio-Rad) for 1 h at room temperature.

Membranes were incubated with primary antibodies against CSF1R overnight at 4 °C, and with β-actin for 3 h at room temperature (Table 2). After washing, appropriate secondary antibodies (anti-rabbit IRDye® 800CW and anti-mouse IRDye® 680RD; LI-COR; 1:10,000) were applied for 1 h at room temperature. Signals were visualized using the LI-COR Odyssey CLx Imaging System (LI-COR Biosciences) (Table 2).

**Table 2:** List of antibodies used for western blotting

Target Ag	Host	Cat #	Manufacturer	Antibody dilution
Primary antibody				
CSF1R monoclonal	Rabbit	ab239079	Abcam	1:700
β-actin	Mouse	MAB1501	Millipore	1:1000
Secondary antibody				
anti-Rabbit IRDye® 800CW	Goat	925–32,211	LI-COR	1:10,000
anti-Mouse IRDye® 680RD	Goat	925–68,070	LI-COR	1:10,000

Catalog number (Cat #), Colony-stimulating factor 1 receptor (CSF1R)

Densitometric analyses of the western blot images were conducted on the Licor Image Studio Lite software. This approach ensures more reliable and reproducible results, as the signal intensity values are unaffected by any image brightness and contrast alteration. Using the profile tool, rectangular boxes were placed around each band to define the region of interest. Subsequently, the background signals were corrected by activating the software’s feature to average all background densities. Finally, by enabling the quantification checkbox in the analysis tab, we obtained the background-corrected signals (optical density) for each CSF1R band. Similarly, the housekeeping gene (beta-actin) level for each blot was quantified. The software automatically computes the signal value by subtracting the product of band area and background intensity from the total measured intensity. The final CSF1R protein levels were recorded following normalization with beta-actin as a reference protein. The normalized values were statistically analyzed to compare CSF1R protein expression levels across experimental conditions.

### Statistical analysis

Data are presented as mean ± standard deviation (SD). Comparisons among neurodegenerative disease groups were conducted using a mixed linear model (*m*0: Density.Mean.perCase ~ ND.Group + Frequency + Sex + (1│case)), where 'Frequency' denotes the number of areas analyzed per case. Multiple testing correction was applied using the Holm method. For comparisons of plaque densities in MS cases, a separate linear model was used (*m*1: Plaque.Density ~ Group + (1│case)), also corrected using Holm adjustment. Additionally, proportions were compared using the function *pairwise.prop.test* from the R stats package. SV2A and synaptophysin densities data were obtained from a previous study by our group [15]. Matched cases from the SV2A and CSF1R datasets and synaptophysin and CSF1R datasets were compared using mean expression across six predefined regions of interest (ROIs) per subject. To test correlation robustness, a bootstrap (n = 10,000´) analysis was performed by simulating 30 matched pairs from normal distributions defined by the observed mean and standard deviation per ROI and group (neurodegenerative disease or control). An empirical p-value was estimated from the proportion of simulated correlations matching the direction and magnitude of the observed result. All statistical analyses were performed in R version 4.5.0 (R Foundation for Statistical Computing, Vienna, Austria).

## Results

### CSF1R-positive microglia density levels in healthy human brains

**Fig. 2:**
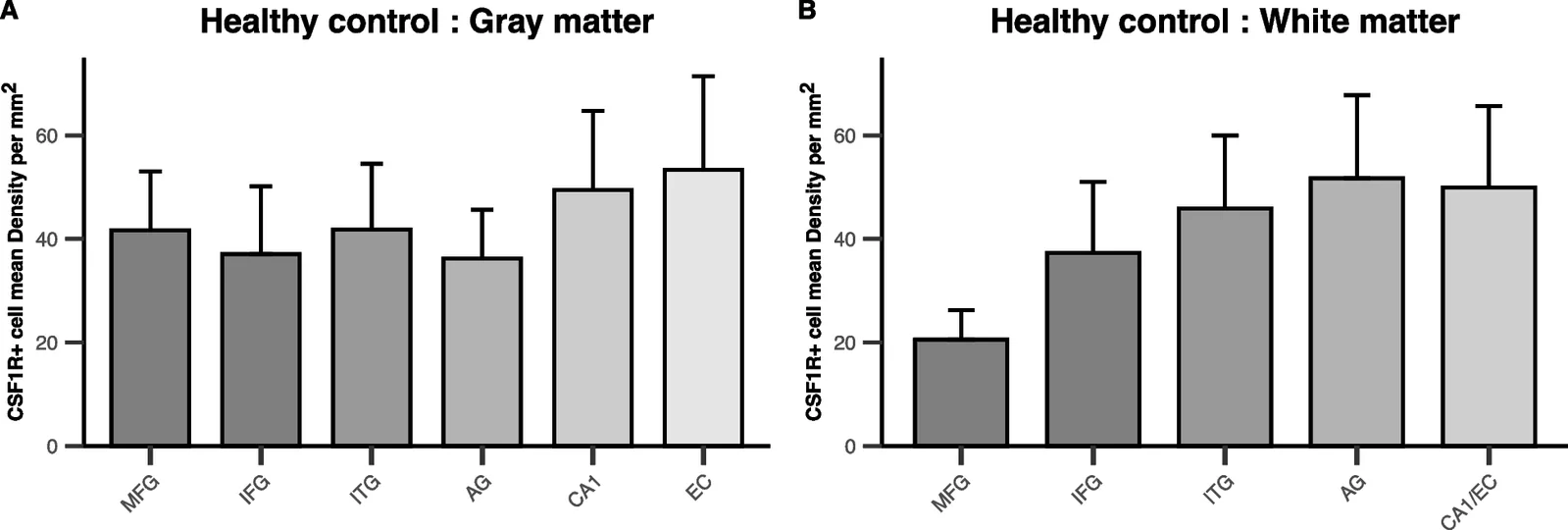
CSF1R-positive (CSF1R +) microglia density in healthy control cases. A and B display low variation in CSF1R RNA cell density (cell/mm^2^) in gray matter and white matter, respectively. Middle frontal gyrus (MFG), Inferior frontal gyrus (IFG), Inferior temporal gyrus (ITG), Angular gyrus (AG), Cornu Ammonis sector 1 of the hippocampus (CA1), Entorhinal cortex (EC)

We mapped CSF1R-positive microglia density in gray and white matter from healthy controls across multiple cortical regions. In gray matter, CSF1R-positive microglia density showed modest regional variation, with lower mean densities in the angular gyrus and inferior frontal gyrus, intermediate values in the middle frontal gyrus and inferior temporal gyrus, and higher values in CA1 and entorhinal cortex. In white matter, densities were lowest in the MFG (the control baseline used for MS comparisons) and were higher across the remaining sampled regions in Fig. 2. No significant ROI-level differences were detected in control gray or white matter (Fig. 2).

### CSF1R-positive microglia density in gray matter in cases with neurodegenerative diseases

We observed disease- and region-specific increases in CSF1R-positive microglia density across the neurodegenerative conditions examined.

**Fig. 3:**
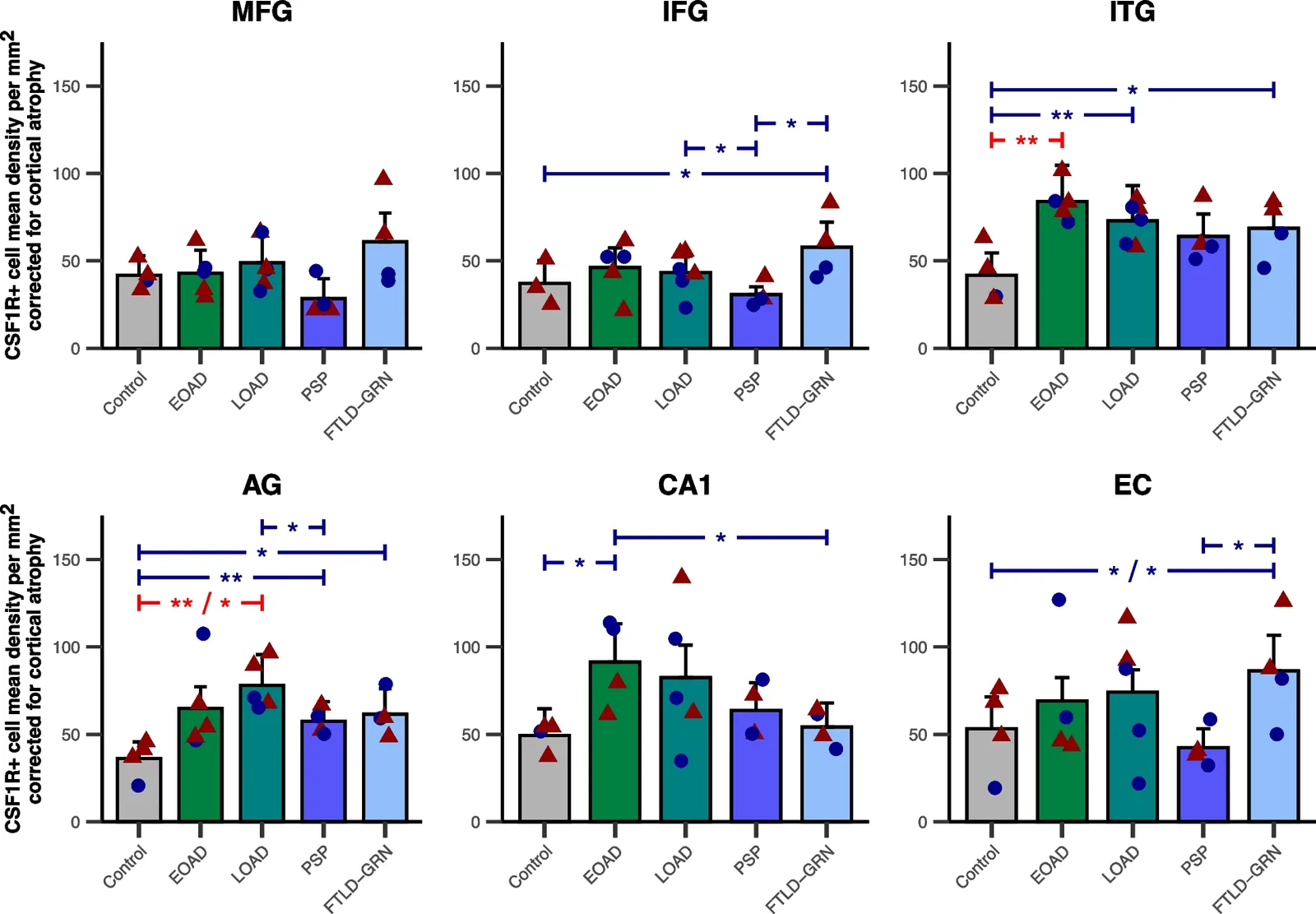
Comparison of: Atrophy-corrected gray-matter CSF1R-positive microglia mean density in the gray matter across different brain regions in neurodegenerative conditions and healthy controls. All values are presented as mean ± SD per group. Statistical significance for mixed-linear model *m*0 (see methods) is indicated as follows: * *p-value* < 0.05, ** *p-value* < 0.01. Red *p*-value bars indicate comparisons that remain significant after Holm correction for multiple pathological conditions. In cases where a second p-value is shown, it reflects the significance of the sex variable in model *m*0. Dark-red triangles represent female subjects, while dark-blue circles represent male subjects. Healthy controls (Control), early-onset Alzheimer’s disease (EOAD), late-onset Alzheimer’s disease (LOAD), progressive supranuclear palsy (PSP), and FTLD-TDP type A due to progranulin mutation (FTLD-GRN), across the middle frontal gyrus (MFG), inferior frontal gyrus (IFG), inferior temporal gyrus (ITG), angular gyrus (AG), hippocampal Cornu Ammonis sector 1 (CA1), and entorhinal cortex (EC)

In EOAD, mapping CSF1R-positive microglia density across six cortical brain regions revealed distinct patterns of microglial activation. The ITG and CA1 exhibited the most pronounced increases compared to healthy controls, although the latter lost statistical significance after correction for multiple comparisons. A region-specific gradient of change was observed, with the MFG and IFG showing the most modest increases in CSF1R-positive microglia density. Although, besides ITG and CA1, no other regions showed statistically significant differences relative to controls (Fig. 3; Table 3).

**Table 3:** Atrophy-corrected CSF1R-positive microglia density changes across neurodegenerative diseases

Brain region	Groups	With cortical atrophy correction			
		**Mean (SD) CSF1R + microglia density (cell/mm**^**2)**^	**% change in CSF1R + microglia density compared to Controls**		***P value (cuncorrected)***
MFG					
	Control	41.7 (11.37)			
	EOAD	43 (13.06)		3.1	0.886
	LOAD	48.9 (16.38)		17.3	0.403
	PSP	28.4 (11.4)		−31.9	0.095
	FTLD-GRN	60.8 (16.56)		45.8	0.079
IFG					
	Control	37.1 (13.08)			
	EOAD	46.3 (11.21)		24.8	0.689
	LOAD	43.3 (9.52)		16.7	0.169
	PSP	30.6 (4.55)		−17.5	0.818
	FTLD-GRN	57.9 (14.15)		56.1	**0.033**
ITG					
	Control	41.8 (12.66)			
	EOAD	84 (20.7)		101.0	**0.002**
	LOAD	73 (20.13)		74.6	**0.009**
	PSP	63.9 (12.86)		52.9	0.052
	FTLD-GRN	68.7 (13.82)		64.4	**0.019**
AG					
	Control	36.2 (9.41)			
	EOAD	64.9 (12.25)		79.3	0.108
	LOAD	78.1 (17.63)		115.7	** < 0.001**
	PSP	57.4 (11.27)		58.6	**0.009**
	FTLD-GRN	61.6 (14.52)		70.2	**0.043**
CA1					
	Control	49.4 (15.3)			
	EOAD	91.3 (21.83)		84.8	**0.018**
	LOAD	82.4 (18.51)		66.8	0.137
	PSP	63.6 (15.97)		28.7	0.239
	FTLD-GRN	54.1 (13.85)		9.5	0.529
EC					
	Control	53.3 (18.13)			
	EOAD	69.2 (13.32)		29.8	0.621
	LOAD	74.1 (12.82)		39.0	0.052
	PSP	42.5 (10.79)		−20.3	0.648
	FTLD-GRN	86.4 (20.23)		62.1	**0.022**

Standard deviation (SD), Middle frontal gyrus (MFG), Early-onset Alzheimer’s disease (EOAD), Late-onset Alzheimer’s disease (LOAD), Progressive supranuclear palsy (PSP), and FTLD-TDP type A due to progranulin mutation (FTLD-GRN), Inferior frontal gyrus (IFG), Inferior temporal gyrus (ITG), Angular gyrus (AG), Hippocampal Cornu Ammonis sector 1 (CA1), Entorhinal cortex (EC). Significant *p*-values (*p* < 0.05) are in bold

In LOAD, the AG showed the greatest increase in CSF1R-positive microglia density, followed by ITG. However, the ITG increases in density lost significance after correction for multiple comparisons. The EC exhibited a near‑significant elevation (Fig. 3; Table 3). As in EOAD, a regional gradient was evident, with MFG and IFG displaying only modest, non‑significant increases.

In PSP, the AG again showed the highest CSF1R-positive microglia density and reached significance before correction, but this significance was lost after adjustment. Notably, the IFG, MFG, and EC trended toward lower CSF1R-positive microglia densities than controls, though none reached statistical significance (Fig. 3; Table 3).

In FTLD‑GRN, CSF1R-positive microglia density increases were more widespread than in other neurodegenerative groups, with AG, ITG, EC, and IFG exhibiting the largest (lost significance after multiple corrections) elevations (Fig. 3). In FTLD‑GRN, CSF1R-positive microglia density increases were more widespread than in other neurodegenerative groups, with AG, ITG, EC, and IFG exhibiting the largest (lost significance after multiple corrections) elevations (Fig. 3; Table 3).

Several comparisons attenuated after Holm correction (see black *p*‑value bars in Fig. 3), but the regional ordering was consistent with the raw estimates. As expected, uncorrected densities showed larger magnitude changes than thickness‑corrected values (Table S1 vs Table 3).

### CSF1R-positive microglia density in white matter in cases with neurodegenerative diseases

**Fig. 4:**
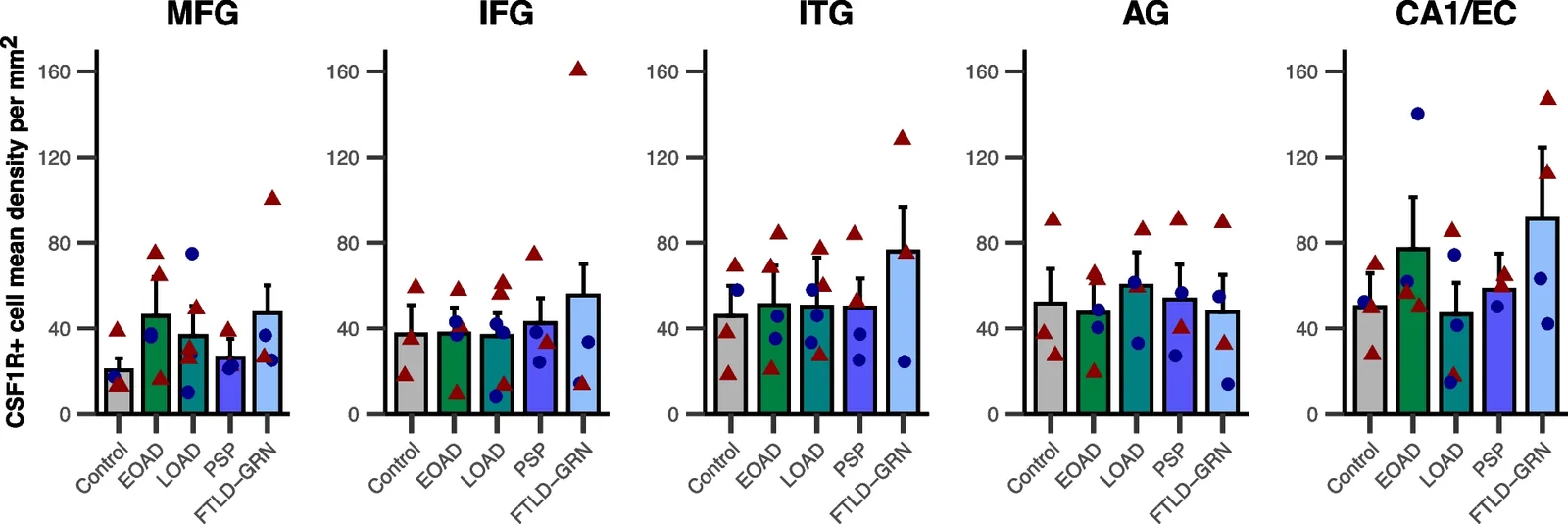
Comparison of: White-matter CSF1R-positive microglia across different brain regions and neurodegenerative diseases. All values are presented as mean ± SD. No statistically significant differences were found in any comparison using the mixed linear model *m*0. Dark-red triangles represent female subjects, while dark-blue circles represent male subjects. Healthy controls (Control), Early-onset Alzheimer’s disease (EOAD), Late-onset Alzheimer’s disease (LOAD), Progressive supranuclear palsy (PSP), and FTLD-TDP type A due to progranulin mutation (FTLD-GRN), across the middle frontal gyrus (MFG), inferior frontal gyrus (IFG), inferior temporal gyrus (ITG), angular gyrus (AG), and hippocampal sector 1 and entorhinal cortex (CA1/EC) combined

In contrast to the prominent increase in CSF1R-positive microglia density observed in cortical gray matter regions, changes in the white matter were milder or undetectable. We did not observe significant differences in CSF1R-positive microglia density in the IFG or AG across any disease condition compared to controls (Fig. 4). Altogether, the pattern observed suggests that microglial alterations are both disease- and region-specific. Except for the AG, FTLD-GRN showed the greatest magnitude of CSF1R-positive microglia density increase relative to controls, with female subjects showing the most prominent increase in density.

### CSF1R-positive microglia density in the gray matter plaques and white matter of multiple sclerosis cases

**Fig. 5:**
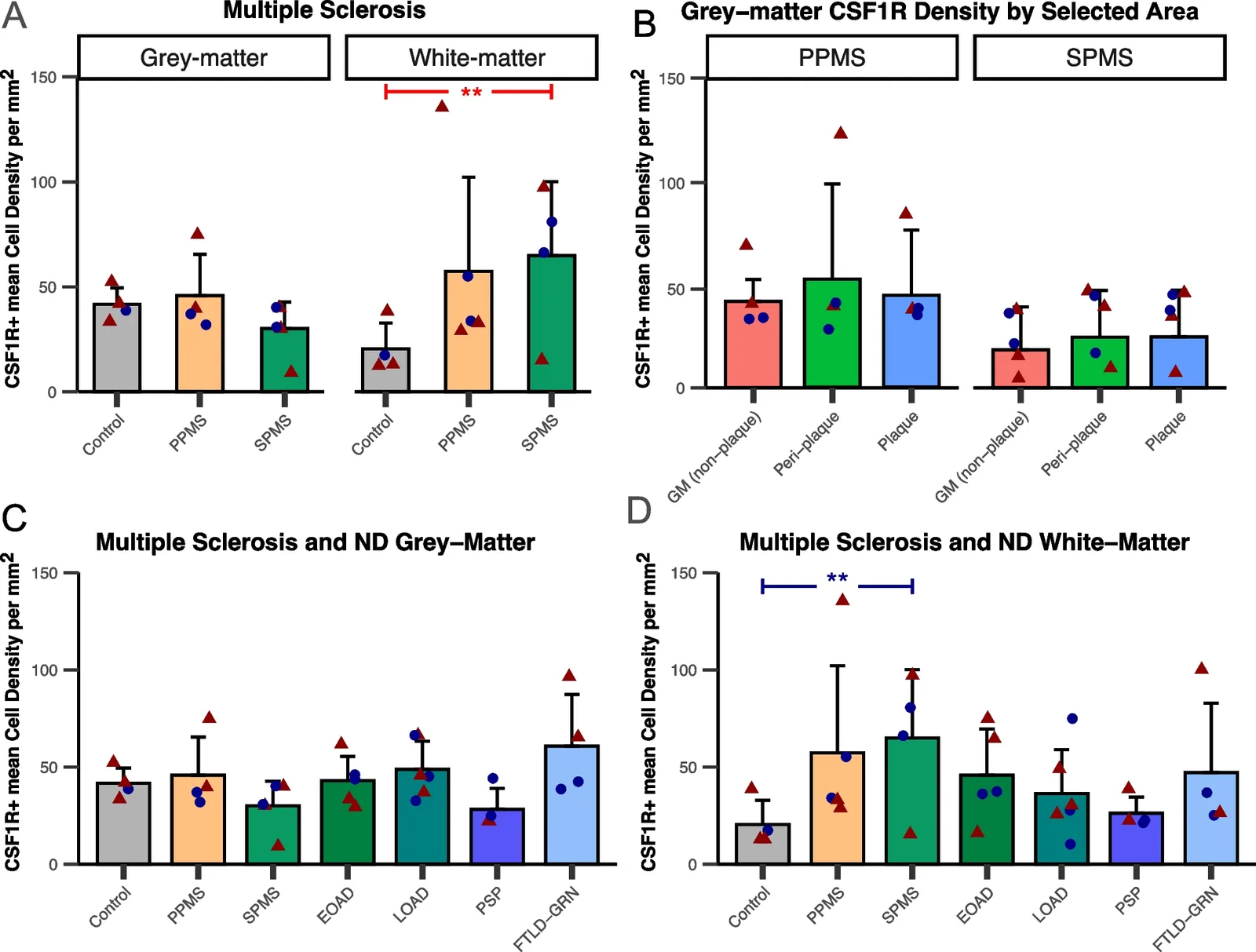
Lesion-level context for CSF1R-positive microglia density in progressive multiple sclerosis (MS) and comparison with neurodegenerative diseases. **A** Mean CSF1R + cell density (cells/mm2) in gray matter (GM) and white matter (WM) from healthy controls (Control), primary progressive MS (PPMS), and secondary progressive MS (SPMS). **B** Mean CSF1R + cell density (cells/mm2) across selected GM compartments in PPMS and SPMS cases, including non-plaque GM, periplaque GM, and plaques. **C** Mean CSF1R + cell density (cells/mm2) in GM from Control, PPMS, SPMS, and neurodegenerative disease groups. **D** Mean CSF1R + cell density (cells/mm2) in WM from Control, PPMS, SPMS, and neurodegenerative disease groups. For the neurodegenerative disease comparisons, values represent the middle frontal gyrus (MFG). Bars represent mean ± SD. Statistical significance is indicated as *p < 0.05, **p < 0.01. Dark-red triangles represent female subjects, and dark-blue circles represent male subjects. Early-onset Alzheimer’s disease (EOAD), late-onset Alzheimer’s disease (LOAD), progressive supranuclear palsy (PSP), and FTLD-TDP type A due to progranulin mutation (FTLD-GRN)

Representative HLA-PLP stained sections used to delineate cortical GM plaques and the corresponding CSF1R RNAscope signal in PPMS and SPMS. CSF1R-positive microglia density was quantified in three gray-matter compartments: demyelinated cortical gray-matter plaques, plaque-adjacent gray-matter penumbra, and distant-appearing gray matter. The plaque-adjacent penumbra was defined as the 500-µm gray-matter region surrounding the plaque border, whereas distant gray matter was sampled from areas at least 3 mm from any plaque. Histologically unaffected white matter was analyzed separately (Fig. S1). In progressive MS, CSF1R-positive microglia density in gray matter remained similar to controls (Fig. 5A; Table 4). Within MS gray matter, CSF1R-positive microglia densities across distant to plaque, penumbral/periplaque, and plaque regions were variable but did not show a significant difference in either PPMS or SPMS (Fig. 5B). In white matter, density was higher in in SPMS than in controls: controls 20.5 (12.36) cells/mm2 vs. SPMS 64.9 (35.27) cells/mm^2^ (+ 216.59%, *p* = 0.005). PPMS and SPMS white-matter densities were not significantly different from each other.

**Table 4:** CSF1R-positive microglia density comparisons in progressive multiple sclerosis gray and white matter

Plaque	**Comparison**	**Mean (SD) in baseline** (cell/mm^2^)	**Mean (SD) in comparative groups** (cell/mm^2^)	**% change in CSF1R + microglia density**	**P value of *m*1**
Gray matter					
	Control vs. PPMS	Control = 41.7 (7.91)	45.9 (19.60)	10.07	0.339
	Control vs. SPMS	Control = 41.7 (7.91)	30.1 (12.64)	−27.82	0.636
	PPMS vs. SPMS	PPMS = 45.9 (19.60)	30.1 (12.64)	−34.42	0.066
White matter					
	Control vs. PPMS	Control = 20.5 (12.36)	57.4 (44.83)	180.00	0.336
	Control vs. SPMS	Control = 20.5 (12.36)	64.9 (35.27)	216.59	**0.005**
	PPMS vs. SPMS	PPMS = 57.4 (44.83)	64.9 (35.27)	13.07	0.894

Primary progressive multiple sclerosis (PPMS), secondary progressive multiple sclerosis (SPMS), and standard deviation (SD). Significant p-values (*p* < 0.05) are in bold

In the broader cross-disease comparison, gray-matter CSF1R-positive microglia density was highest in FTLD-GRN rather than in MS. FTLD-GRN gray matter was higher than control gray matter (*p* = 0.0132), PPMS gray matter (*p* = 0.0416), SPMS gray matter (*p* = 0.0261), LOAD gray matter (*p* = 0.0432), and PSP gray matter (*p* = 0.0384) (Fig. 5C/D**)**.

### CSF1R protein levels in healthy control and AD tissues were measured by Western blot

**Fig. 6:**
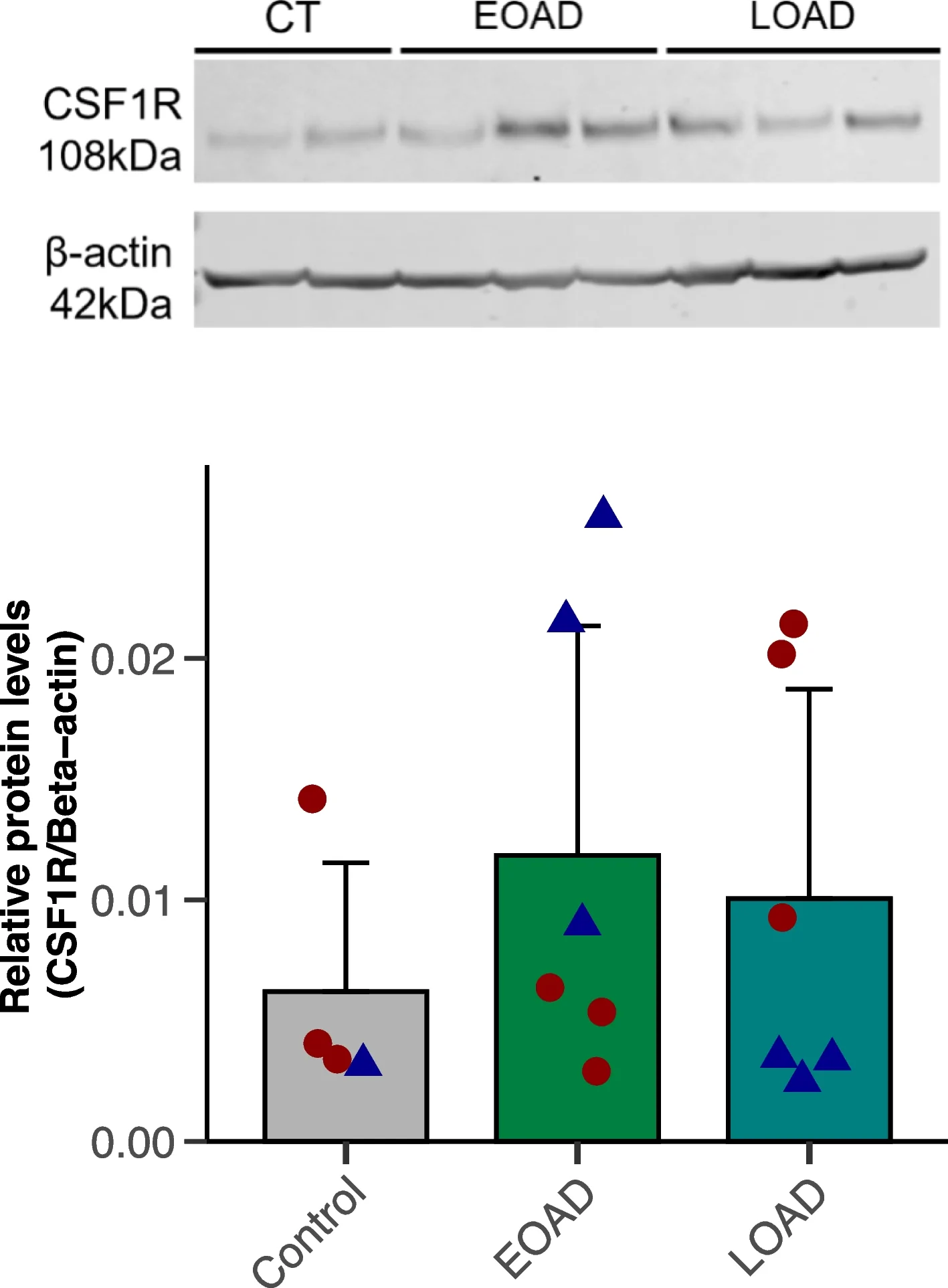
Western blot quantification of CSF1R protein levels. Immunoblot image showing CSF1R (108 kDa) and β-actin (42 kDa) in the inferior frontal gyrus from healthy controls (Control), early-onset Alzheimer’s disease (EOAD), and late-onset Alzheimer’s disease (LOAD) cases. No significant differences in CSF1R protein levels were observed between EOAD and LOAD cases compared to Control cases in the inferior frontal gyrus. All values are presented as mean ± SD. Dark-red triangles represent female subjects, and dark-blue circles represent male subjects

We performed Western blot to directly measure CSF1R protein levels in the IFG of healthy controls, EOAD, and LOAD (Table 1). Similarly to the corrected histological assessment, CSF1R levels are graphically increased in the IFG of EOAD (*p* = 0.4079) and LOAD (*p* = 0.7141) cases compared to controls, but statistically not significant (Fig. 6 and Fig. S2).

### Association of CSF1R-positive microglia and SV2A and synaptophysin densities in gray matter in neurodegenerative diseases

In our earlier work, we mapped synaptic density using the presynaptic markers SV2A and synaptophysin across the same brain regions, neurodegenerative conditions and largely the same post‑mortem we now use to quantify CSF1R-positive microglia density [15]. To determine whether CSF1R‑positivity cell density increases, as a marker of microglia activation, tracks with changes in synaptic density, we regressed CSF1R-positive microglia against SV2A and synaptophysin densities (separately) across all six ROIs in matched cases. A negative slope would suggest that heightened CSF1R activity accompanies synaptic depletion, whereas a positive slope could point to a CSF1R-positive microglial response associated with relative synaptic preservation.

**Fig. 7:**
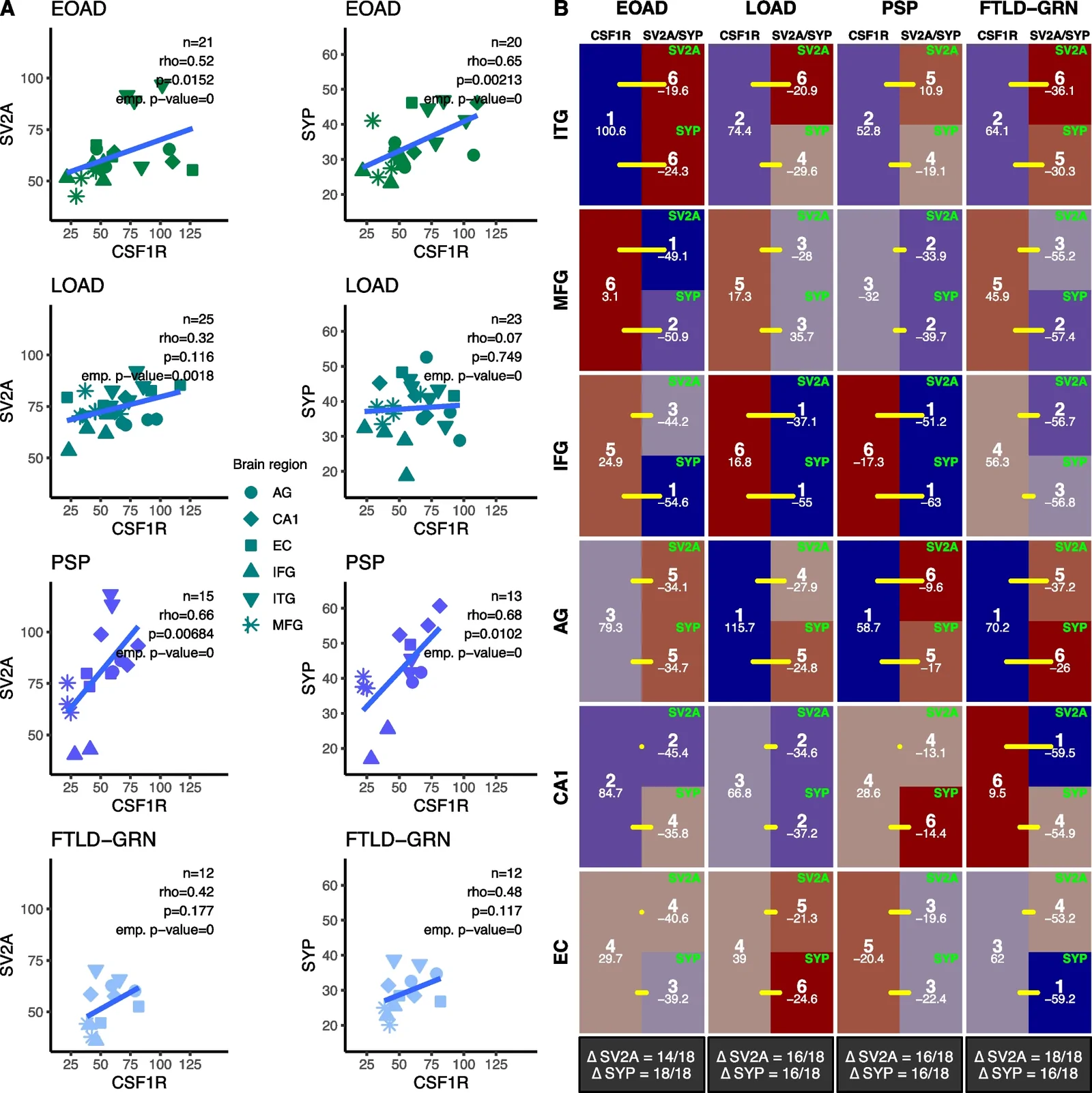
Correlation between CSF1R-positive microglia and presynaptic markers across brain regions in neurodegenerative diseases and rank change analysis. **A** Scatter plots show the relationship of CSF1R against SV2A and synaptophysin (SYP) densities across six regions of interest (ROIs) for each diagnostic group: early-onset Alzheimer’s disease (EOAD), late-onset Alzheimer’s disease (LOAD), progressive supranuclear palsy (PSP), and frontotemporal lobar degeneration with GRN mutation (FTLD-GRN). Each point represents the mean density in each ROI for a matched case between this study and the SV2A/SYP study [15]; marker shapes indicate specific brain regions. Linear regression lines are shown in blue. Sample size (n), Spearman correlation coefficient (rho), Spearman correlation p-value (p), and empirical *p*-values based on bootstrap analysis are indicated in each plot. **B** Rank-based comparison of CSF1R against SV2A and SYP levels across six brain regions (ITG, MFG, IFG, AG, CA1, EC) in four neurodegenerative disease groups (EOAD, LOAD, PSP, FTLD-GRN). For each group, regions were independently ranked from 1 (highest) to 6 (lowest) based on mean density of each marker. Yellow bars indicate the absolute difference in ranks between CSF1R and SV2A/SYP per region. A total Δ-rank score (bottom row) represents the sum of all absolute rank differences across regions; a maximum possible Δ-rank of 18 would indicate an almost perfectly inverse regional pattern between the markers

Across all neurodegenerative groups, CSF1R-positive microglia and SV2A densities were positively correlated (Fig. 7A). The association was strongest in PSP (ρ = 0.66, *p* = 0.0069; empirical *p* = 0) and weakest in LOAD (ρ = 0.32, *p* = 0.116; empirical *p* = 0.0018). Positive slopes were seen in synaptophysin too, except for LOAD where the slope was almost flat (Fig. 7A). Ranking the magnitude of change relative to controls (Fig. 7B) provided a complementary perspective. In every disease condition, the two brain regions showing the *smallest increases* in CSF1R-positive microglia density corresponded to one of the three regions with the *largest decreases* in SV2A and synaptophysin—except for CA1 in FTLD-GRN, when comparing CSF1R with synaptophysin (Fig. 7B). Conversely, at the other end of the spectrum, in all conditions the region with the *largest increase* in CSF1R-positive microglia density corresponded to one of the two regions with the *smallest decreases* in SV2A and synaptophysin, except for the angular gyrus in LOAD when comparing CSF1R with SV2A.

## Discussion

We created a cross-disease, region-resolved map of CSF1R-positive microglia in human brain and found that CSF1R changes depend strongly on disease, region, and tissue compartment. Neurodegenerative diseases showed selective gray-matter increases, with distinct regional signatures across EOAD, LOAD, PSP, and FTLD-GRN and minimal white-matter change. Progressive MS did not show gray-matter CSF1R-positive microglial increase in the sampled non-plaque, periplaque, or plaque regions, whereas SPMS showed increased white-matter CSF1R-positive density relative to controls. These data provide histological context for interpreting future CSF1R-PET across disease settings.

Focusing on the neurodegenerative groups, we then directly compared CSF1R-positive microglia density with SV2A and synaptophysin synaptic densities reported previously [15], leveraging adjacent sections from mostly the same cases. Remarkably, CSF1R microglial elevation often coincided with areas of relative synaptic preservation and vice versa. This region-specific dissociation reinforces experimental observations that certain microglial states may serve protective or compensatory functions and in humans CSF1R-positive microglia may overlap with microglial states previously associated with tissue maintenance or repair. This nuanced view is relevant as CSF1R PET ligands are in development to image microglia in vivo: interpreting these signals must account for their potential protective as well as potentially detrimental roles, which may vary across regions and diseases.

### CSF1R-positive microglia density in neurodegenerative diseases: modest, region-specific changes

Although many histological and PET investigations have reported increased activated microglia and their association with cognitive decline in AD and other neurodegenerative conditions—for example, PET imaging with the microglial tracer ^11^C-PK11195, which binds to TSPO (a marker upregulated in activated but not resting microglia) [25–32]—only a few studies have examined CSF1R-positive cell density in human brains. Those studies, focused mainly on LOAD, compared individuals with and without neuropathology but were not designed to provide quantitative multi-region mapping. Autoradiography and gene expression studies have demonstrated elevated CSF1R levels in postmortem AD brain tissue compared with controls [5, 10, 33]. Histological studies remain limited but indicate substantial overlap between CSF1R and CD68 labeling within the same microglia, indirectly suggesting that CSF1R-positive microglia are enriched around amyloid plaques and in pathology-rich regions such as the hippocampus, entorhinal cortex, and temporal cortices, as shown in prior CD68 studies of AD brains [9, 34]. Additional histological investigations using CD68 and HLA-DR have reported robust microglial activation in frontotemporal cortices across FTLD subtypes, including FTLD-GRN [34–38], which indirectly supports our findings. While previous histological studies have not specifically quantified CSF1R expression in PSP, postmortem analyses using markers such as HLA-DR have documented elevated microglial activation in both cortical and subcortical PSP regions [39]. In contrast, our PSP cases showed the most modest CSF1R-related changes among all conditions tested. Finally, female sex appeared to partially drive the LOAD–control differences. Although our sample was underpowered for definitive sex-specific analysis, these findings are consistent with growing evidence for sex-linked pathways in AD pathogenesis [40, 41].

### Exploratory alignment with presynaptic markers: can CSF1R-positive microglia protect against synaptic loss in neurodegenerative diseases?

In our study, we included sporadic EOAD cases with onset < 65 years and analyzed them separately from LOAD, justified by evidence that the two forms diverge in cortical atrophy and clinical course [42, 43]. Although no widespread differences emerged, EOAD showed significantly higher CA1 CSF1R-positive microglia density versus controls, a pattern not statistically significant in LOAD, while AG was relatively preserved in EOAD yet markedly affected in LOAD (Fig. 3). Imaging work, however, reports the opposite trend: greater AG atrophy in EOAD and greater CA1 loss in LOAD [44, 45]. This inversion may point to compensatory neuroprotective responses, such as CSF1R-driven microglial proliferation that selectively buffers degeneration [11, 46]. To further examine this possibility, we leveraged our previous work on SV2A and synaptophysin mapping in the same conditions and regions [15]. We found that EOAD exhibited a greater decline in SV2A density than LOAD within the AG, while LOAD showed a greater decline in synaptophysin density within CA1. Similarly, in FTLD-GRN, the regions with the largest increases in CSF1R-positive microglia (ITG and AG) coincided with the mildest declines in SV2A and synaptophysin density. Notably, in PSP the only region showing a significant increase in CSF1R-positive microglia compared with controls was the AG. In our earlier work, the AG was also the region with the mildest decrease in SV2A and the second-mildest decrease in synaptophysin density, consistent with the relative preservation of posterior cortices compared with frontal regions in PSP [47]. Conversely, the IFG, which is among the most affected cortical regions in PSP [18], showed the most severe loss of SV2A and synaptophysin in our previous study, yet exhibited the least change in CSF1R-positive microglia.

In sum, a direct comparison of the magnitude of change in CSF1R-positive microglia density and SV2A synaptic density across all neurodegenerative conditions examined revealed an intriguing pattern: regions with the largest increases in CSF1R-positive cell density often exhibited the smallest decreases in pre-synaptic protein densities. This suggests a possible protective role for CSF1R-positive microglia in maintaining synaptic integrity. To further evaluate this relationship, we performed regression analyses comparing CSF1R-positive microglia and pre-synaptic protein densities within parallel sections from the same cases which confirmed the trend especially in EOAD and PSP, and bootstrap analyses reinforced the robustness of these findings and suggest that LOAD and FTLD-GRN follow the same direction.

To our knowledge, relatively few studies have directly compared microglial populations (specifically using CSF1R markers) and synaptic loss in human tissue or CSF1R and pre-synaptic markers animal models. Most prior work in humans has assessed microglial activation using PET imaging of TSPO, which identifies microglia in different activation states and does not specifically isolate CSF1R-positive microglia. Direct neuropathological studies in experimental models that relate microglia to synaptic density have typically relied on markers such as IBA1, CD68, HLA, or allograft inflammatory factor-1, and generally report negative correlations where microglial activation increases where synaptic loss is most severe [1, 48, 49].

Interestingly, studies in AD and tauopathy models have yielded mixed results: CSF1R inhibition can reduce neuroinflammation and improve outcomes in some contexts [10–12], but CSF1R blockade also results in microglial depletion, which can disrupt healthy synapse maintenance and remodeling [13, 14]. Moreover, recent molecular transcriptomic studies, as well as experiments in conditional knockout models, highlight that CSF1R signaling is required for microglial survival and homeostatic function, including pruning of dysfunctional synapses and support for synaptic maintenance [13, 50]. These findings underscore the complexity and context-dependence of microglial roles in neurodegeneration and may explain discrepancies between our findings in post-mortem human tissue and observations from preclinical models.

Taken together, our data suggest that CSF1R-positive microglia may mark a microglial response that differs from purely deleterious neuroinflammatory activation, contrasting with the more toxic, pro-inflammatory microglial phenotypes emphasized in earlier studies. The net effect of microglia on synaptic integrity likely depends on context, activation state, regional vulnerability, and disease stage, rather than on CSF1R expression alone [11, 51, 52]. We emphasize that this analysis was exploratory and cross-sectional; it does not establish causality or a uniform “protective” microglial phenotype. Nonetheless, in human ND tissue, higher CSF1R-positive microglia density can coincide with relative preservation of presynaptic markers, a pattern that merits prospective, multimodal confirmation.

### CSF1R-positive microglia density in multiple sclerosis: compartment-specific white-matter signal with relative gray-matter quiescence

In progressive MS, our findings were compartment-specific rather than uniformly elevated across MS tissue. CSF1R-positive density was not significantly increased in gray matter compared with controls. Within the MS gray matter, non-plaque, periplaque, and plaque compartments were variable but did not show a significant compartment effect. By contrast, white matter showed the clearest increase in CSF1R-positive microglia, reaching significance in SPMS (+ 216.59%, *p* = 0.005) and showing a numerically higher but more variable increase in PPMS (+ 180.00%, *p* = 0.336). This gray-matter result is biologically plausible. Histological lesion activity in MS is defined in large part by the presence and distribution of macrophages/microglia and by evidence of ongoing myelin degradation [53]; therefore, a demyelinated cortical plaque identified by HLA-PLP does not necessarily represent an active, CSF1R-rich lesion at death. Cortical MS lesions, particularly in chronic/progressive disease, can be demyelinated while showing relatively sparse parenchymal inflammatory infiltrates and fewer macrophages/microglia than active white-matter plaques, which we did not have available. Prior pathological studies have described reduced inflammation in cortical MS lesions despite neuroaxonal injury, and widespread cortical demyelination in progressive MS that can coexist with diffuse white-matter injury [54, 55]. In addition, cortical pathology may be shaped by meningeal inflammation and soluble inflammatory factors rather than by dense local accumulation of CSF1R-positive microglia within the sampled cortical parenchyma [56, 57].

The white-matter increase, particularly in SPMS, aligns with the dominant pathology of progressive MS. Progressive MS is associated with diffuse injury in normal-appearing white matter, chronic inactive plaques, and slowly expanding or smoldering lesions in which activated microglia/macrophages can persist at lesion edges and contribute to axonal injury [55, 57–60]. MRI-pathology studies further show that chronic active lesions with iron-laden activated microglia/macrophages at the lesion rim are associated with more severe disease and disability [61]. Therefore, a white-matter CSF1R signal may reflect diffuse microglial activation and/or enrichment of chronic active lesion biology outside the histologically defined cortical plaque core.

CSF1R-pathway literature also supports this interpretation but warrants caution. Hagan et al. reported upregulation of CSF1R and CSF1 in progressive MS tissue and showed that modulation of CSF1R signaling affected microglial/macrophage responses in experimental demyelination [6]. However, CSF1R is a survival and proliferation pathway for myeloid cells, not a single activation-state marker. Active MS lesions show loss of homeostatic microglial signatures and heterogeneous activation programs [62, 63]. Therefore, higher CSF1R-positive density in white matter could be interpreted as evidence of altered myeloid/microglial compartment biology in progressive MS. The PPMS/SPMS pattern should also be interpreted conservatively. PPMS and SPMS share core progressive pathological mechanisms, and differences are often quantitative and influenced by lesion stage, disease duration, sampling site, meningeal inflammation, and white-matter lesion burden rather than by clinical subtype alone [58–60]. In our small cohort, SPMS reached significance in white matter while PPMS did not; however, the PPMS mean was also elevated and highly variable and the sample size is small.

Although we strived to conduct this study using rigorous and unbiased sampling and analysis procedures and well-characterized, high-quality tissue, it is not without limitations. Variability in postmortem interval, along with potential protein and RNA degradation, may have influenced the interpretation of our histological findings. The relatively small sample size (n = 4–5 cases per group) also increases susceptibility to outliers, particularly given interindividual differences in RNA quality and preservation. Nonetheless, we implemented measures to ensure high standards of tissue integrity for all included cases and validated our findings using Western blotting. Finally, when comparing the magnitude of changes in CSF1R-positive cell density with SV2A and synaptophysin densities, we included values even if they were not statistically significant relative to controls. Our intention was to explore potential relationships that could support—or challenge—our hypothesis and thereby inform future studies, rather than to draw absolute conclusions about a possible protective role of CSF1R-positive microglia in neurodegenerative diseases. We did not include a unified regional pathology-burden covariate across neurodegenerative diseases because the relevant histopathological features differ by disease and stage; therefore, the presynaptic-marker analyses should be interpreted as exploratory and potentially confounded by regional disease burden. Another limitation of this study is that analyses were restricted to selected cortical regions and do not capture CSF1R-positive microglial heterogeneity in deep gray nuclei or spinal cord regions, particularly relevant to MS pathology. Reliable cause-of-death information was not available for most cases, because medical records at the time of death were not accessible and family-reported causes were not considered sufficiently reliable for analysis. In addition, we did not have paired CSF1R-PET and autopsy tissue from the same individuals; therefore, the present histological densities provide spatial context for CSF1R-PET interpretation but cannot be used as a direct quantitative calibration of PET signal intensity.

In conclusion, our region- and disease-specific mapping of CSF1R-positive microglia density provides a reference for interpreting in vivo CSF1R PET imaging across neurological disorders. The neurodegenerative cohorts showed selective gray-matter increases, whereas progressive MS showed no gray-matter increase but did show elevated white-matter CSF1R-positive density in SPMS. These compartment-specific findings suggest that CSF1R-PET interpretation will need to account for regional vulnerability, tissue compartment, lesion context, and disease stage. The exploratory alignment between higher CSF1R-positive density and relative preservation of presynaptic markers supports, but does not prove, a possible protective or compensatory CSF1R-microglial state in neurodegenerative disease. Prospective multimodal studies will be required to test this hypothesis directly.

## Supplementary Information

Supplementary Material 1.

## Data Availability

De‑identified per‑ROI CSVs (CSF1R densities), per‑plaque MS data, region‑matched SV2A/SYP summaries, and analysis scripts will be deposited in an open repository upon acceptance; until deposition, data and code are available from the corresponding author on reasonable request.

## Abbreviations

AD: Alzheimer’s disease
AG: Angular gyrus
CA1: Cornu Ammonis 1 (hippocampus)
CI: Confidence interval
CSF1: Colony-stimulating factor 1
CSF1R: Colony-stimulating factor 1 receptor
EC: Entorhinal cortex
EOAD: Early-onset Alzheimer’s disease
FTLD-GRN: Frontotemporal lobar degeneration with TDP-43 inclusions due to GRN mutations
GM: Gray matter
HLA-DR: Human leukocyte antigen-DR isotype
IFG: Inferior frontal gyrus
Iba-1: Ionized calcium-binding adapter molecule 1
ITG: Inferior temporal gyrus
LOAD: Late-onset Alzheimer’s disease
MFG: Middle frontal gyrus
MS: Multiple sclerosis
NBB: Netherlands Brain Bank
ND: Neurodegenerative disease(s)
PET: Positron emission tomography
PMI: Postmortem interval
PPMS: Primary progressive multiple sclerosis
PSP: Progressive supranuclear palsy
QC: Quality control
ROI: Region of interest
RNAscope: RNA in situ hybridization assay (brand name)
SD: Standard deviation
SPMS: Secondary progressive multiple sclerosis
SV2A: Synaptic vesicle protein 2A
SYP: Synaptophysin
TSPO: 18-kDa translocator protein
WB: Western blot
WM: White matter
