# Supplementary material for: Regional mapping of CSF1R-positive microglia in neurodegenerative diseases and progressive MS, with exploratory presynaptic marker analyses
Source: J Neuroinflammation. 2026 Jul 4;23:320. doi: 10.1186/s12974-026-03945-6 (PMC13602470; doi:10.1186/s12974-026-03945-6)
Supplement: Supplementary file 1 [file 12974_2026_3945_MOESM1_ESM.pdf]

SUPPLEMENTARY TABLE AND FIGURES

Regional mapping of CSF1R-positive microglia in neurodegenerative diseases and progressive MS, with exploratory presynaptic marker analyses

Table S1. Percentage (%) of change in CSF1R-positive cell density (cell/µm²) without cortical atrophy correction between healthy controls and each neurodegenerative disease condition across the examined brain regions.

Brain region	Groups	Without cortical atrophy correction		
		**Mean (SD) CSF1R+ cell density** (cell/mm²)	**% change in CSF1R+ cell density compared to Controls**	***P value***
MFG				
	Control	41.7 (11.37)		
	EOAD	60.2 (18.31)	44.4	0.130
	LOAD	58.1 (19.45)	39.3	0.139
	PSP	38.1 (15.33)	-8.6	0.549
	FTLD-GRN	102.6 (27.95)	146.0	**0.013**
IFG				
	Control	37.1 (13.08)		
	EOAD	81.2 (19.66)	118.9	0.112
	LOAD	71 (15.6)	91.4	**0.009**
	PSP	67.9 (10.09)	83.0	**0.035**
	FTLD-GRN	126.4 (30.88)	240.8	**0.002**
ITG				
	Control	41.8 (12.66)		
	EOAD	90.2 (22.25)	115.8	**0.001**
	LOAD	85.3 (23.53)	104.1	**0.003**
	PSP	63.2 (12.7)	51.2	0.056
	FTLD-GRN	98.6 (19.83)	135.9	**0.003**
AG				
	Control	36.2 (9.41)		
	EOAD	88.2 (16.65)	143.6	**0.040**
	LOAD	95.9 (21.66)	164.9	**<0.001**
	PSP	56.1 (11)	55.0	**0.011**
	FTLD-GRN	83.4 (19.65)	130.4	**0.010**
CA1				
	Control	49.4 (15.3)		
	EOAD	130.6 (31.2)	164.4	**0.004**
	LOAD	100.8 (22.64)	104.1	0.077
	PSP	63.7 (15.99)	28.9	0.238
	FTLD-GRN	84 (21.5)	70.0	**0.019**
EC				
	Control	53.3 (18.13)		
	EOAD	94.4 (18.17)	77.1	0.314
	LOAD	78.8 (13.64)	47.8	**0.040**
	PSP	45.9 (11.66)	-13.9	0.818
	FTLD-GRN	116 (27.17)	117.6	**0.007**

Standard deviation (SD), Middle frontal gyrus (MFG), Early-onset Alzheimer's disease (EOAD), Late-onset Alzheimer's disease (LOAD), Progressive supranuclear palsy (PSP), and FTLD-TDP type A due to progranulin mutation (FTLD-GRN), Inferior frontal gyrus (IFG), Inferior temporal gyrus (ITG), Angular gyrus (AG), Hippocampal Cornu Ammonis sector 1 (CA1), Entorhinal cortex (EC). Significant p-values ($p < 0.05$) are in bold.

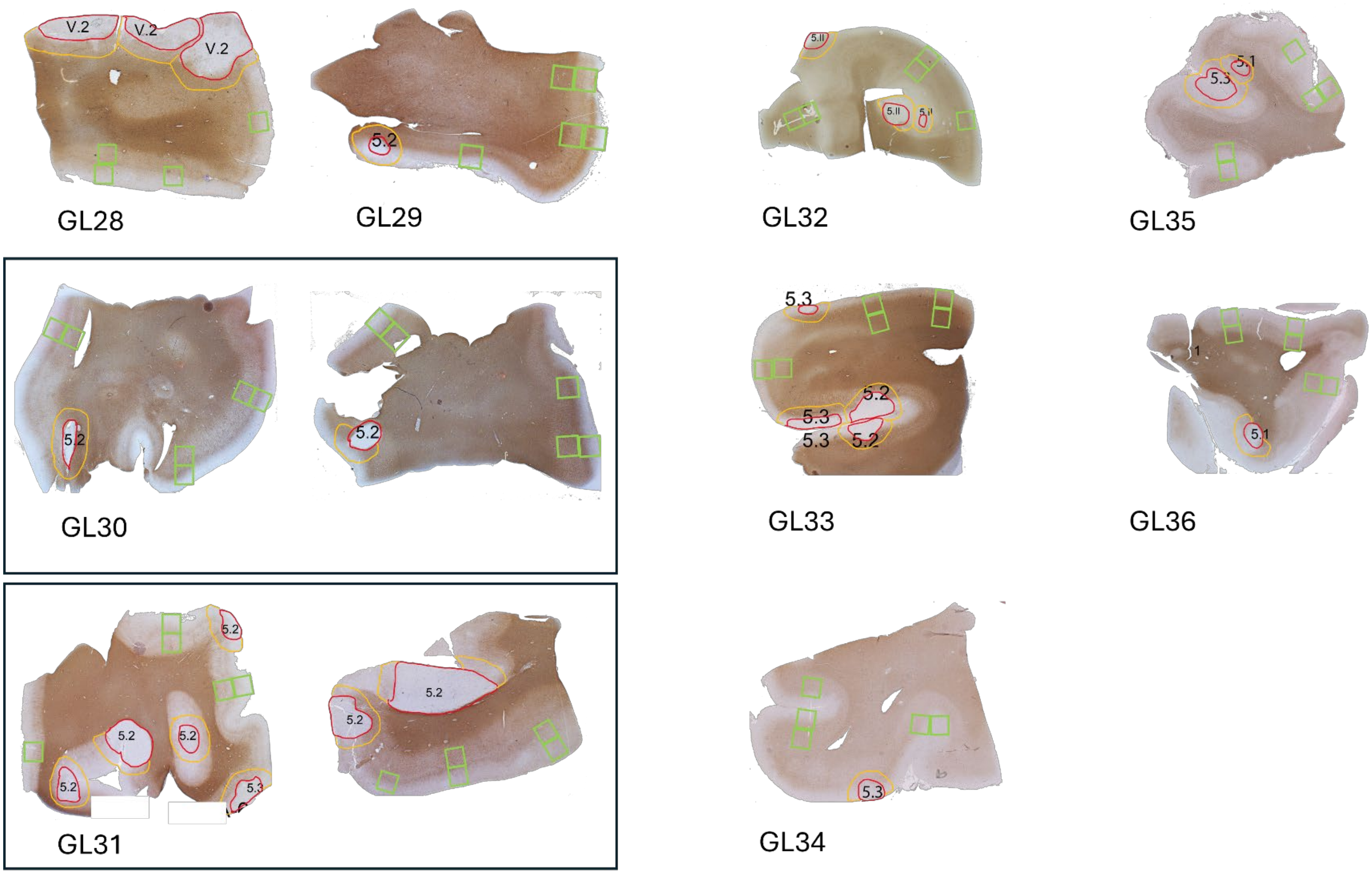

Fig. S1. Overview of HLA-PLP stained multiple sclerosis tissue sections used for lesion annotation. Low magnification whole section images of HLA-PLP stained multiple sclerosis tissue sections from the cases listed in Table 1b. Annotated regions indicate plaques selected for histological assessment and quantitative analysis. Red contours delineate demyelinated plaque areas, yellow contours indicate the surrounding penumbral/periplaque regions, and green boxes mark sampled non lesional reference regions. Case identifiers are shown below each section. The plaque numbers and Roman numerals shown on the images were original identifiers present on the digitized slides received from NDD. These labels indicate histological plaques corresponding to lesions observed by imaging and were retained only for orientation. They do not denote lesion subtype, severity, anatomical hierarchy, or any variable used in the analyses.

Gel 1			CT			EOAD				
Well no	**I**	**II**	**III**	**IV**	**V**	**VI**	**VII**	**VIII**	**IX**	**X**
Cases	RIPA	Ladder	GL15	GL17	GL3	GL9	GL24	GL18	RIPA	RIPA
Lysate loaded	40 µL	07 µL	41.2 µL	41.2 µL	41.2 µL	41. µL	41.2 µL	41.2 µL	40 µL	40 µL
Protein concentration			25ug	25ug	25ug	25ug	25ug	25ug		
Primary Antibody	CSF1R (1:700), Anti-CSF-1-R antibody [EPR20634], Monoclonal, ab239079									
Secondary antibody	800CW (1:10000), Goat anti-Rabbit									
Primary Antibody	Beta-Actin (1:1000), anti-Mouse, MAB1501 Millipore Actin									
Secondary antibody	680RD (1:10000), Goat anti-Mouse									

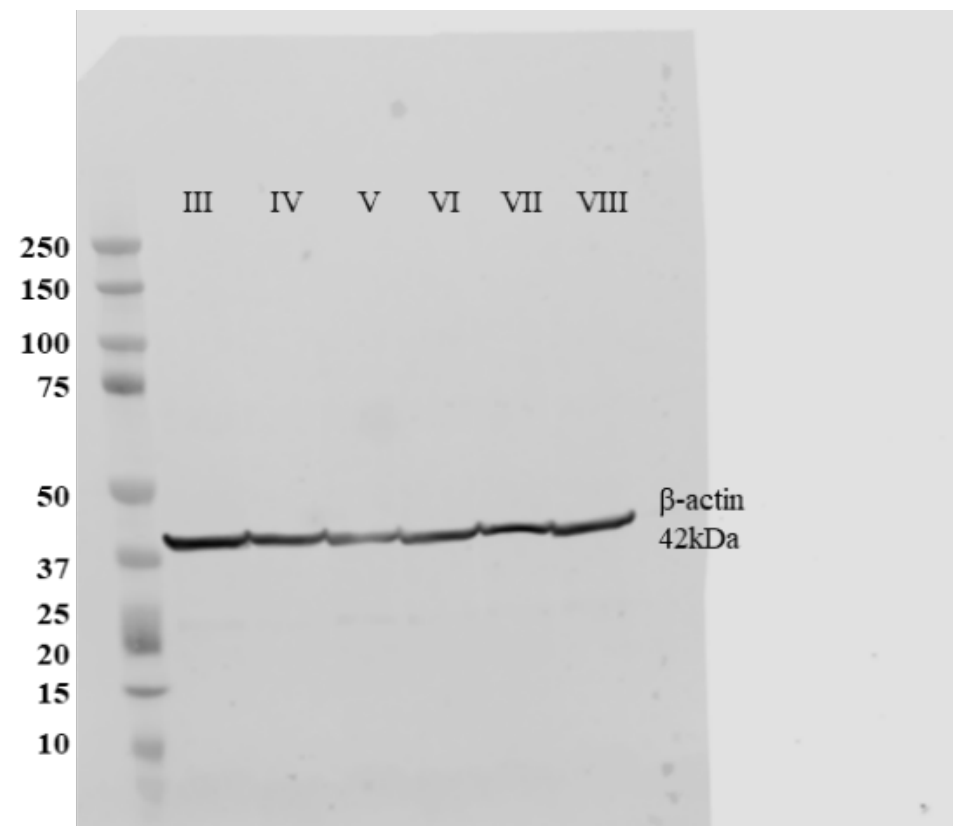

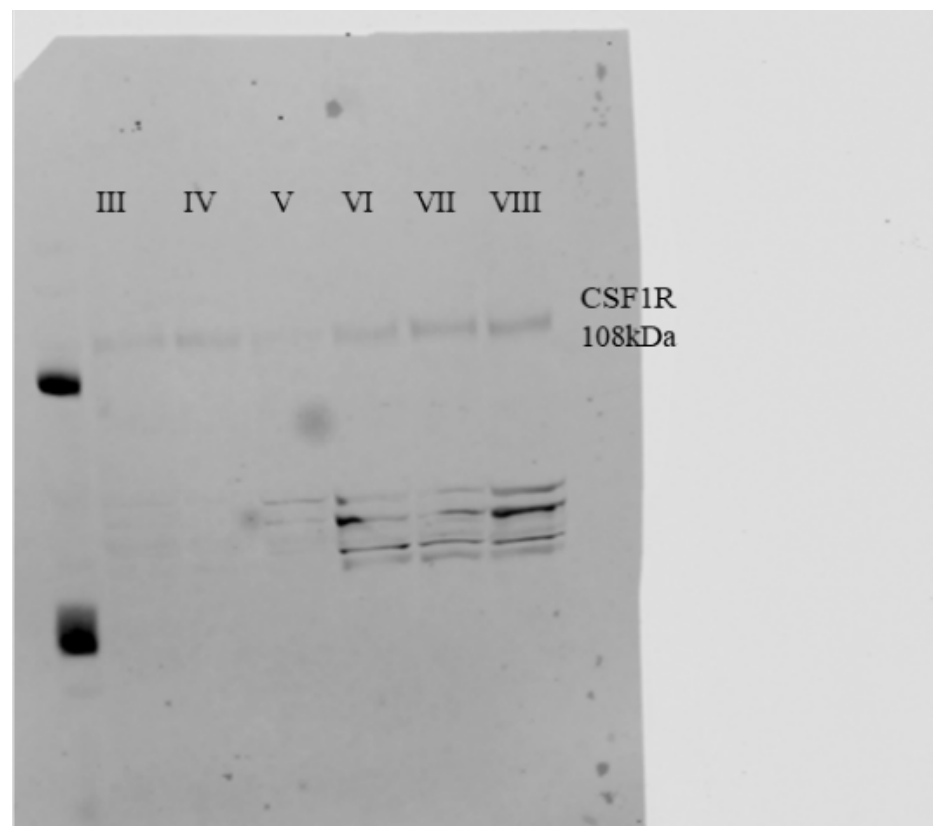

Gel 3		CT		EOAD			LOAD			
Well no	**I**	**II**	**III**	**IV**	**V**	**VI**	**VII**	**VIII**	**IX**	**X**
Cases	Ladder	GL15	GL3	GL25	GL13	GL1	GL26	GL16	GL4	RIPA
Lysate loaded	07 µL	41.2 µL	41.2 µL	41.2 µL	41.2 µL	41.1 µL	41.2 µL	41.1 µL	41.2 µL	40 µL
Protein concentration		25ug	25ug	25ug	25ug	25ug	25ug	25ug	25ug	
Primary Antibody	CSF1R (1:700), Anti-CSF-1-R antibody [EPR20634], Monoclonal, ab239079									
Secondary antibody	800CW (1:10000), Goat anti-Rabbit									
Primary Antibody	Beta-Actin (1:1000), anti-Mouse, MAB1501 Millipore Actin									
Secondary antibody	680RD (1:10000), Goat anti-Mouse									

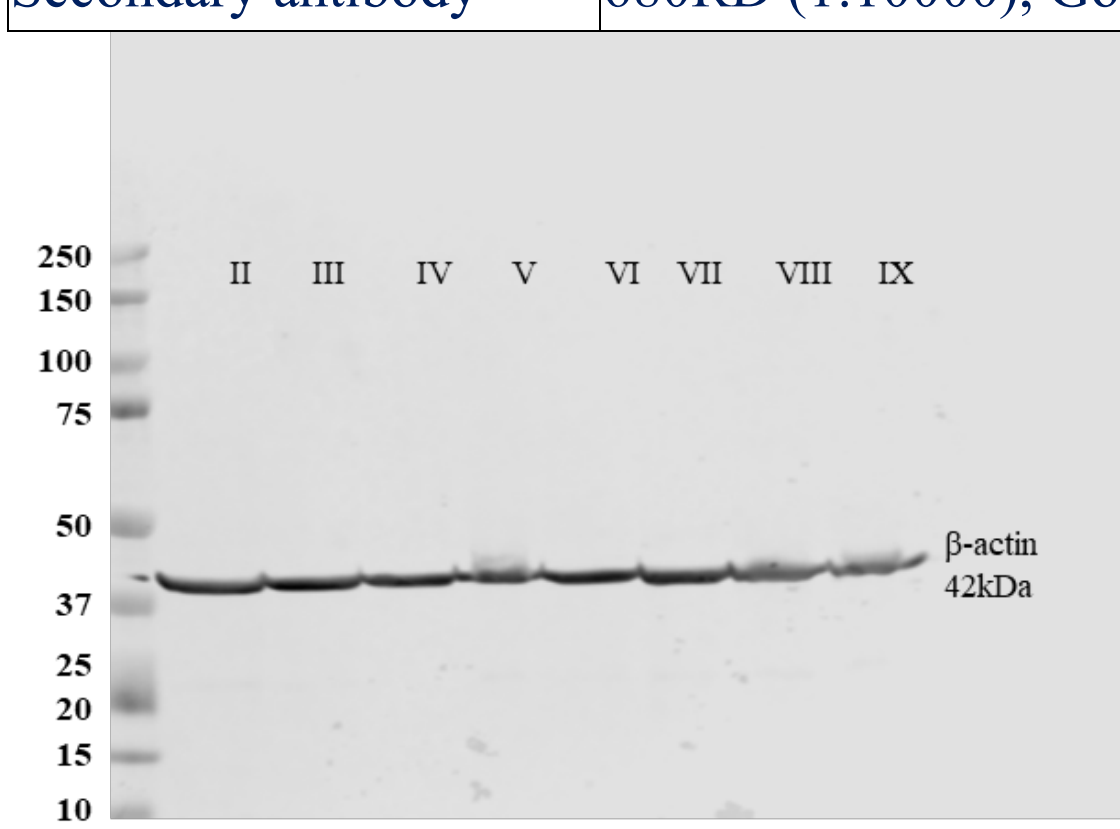

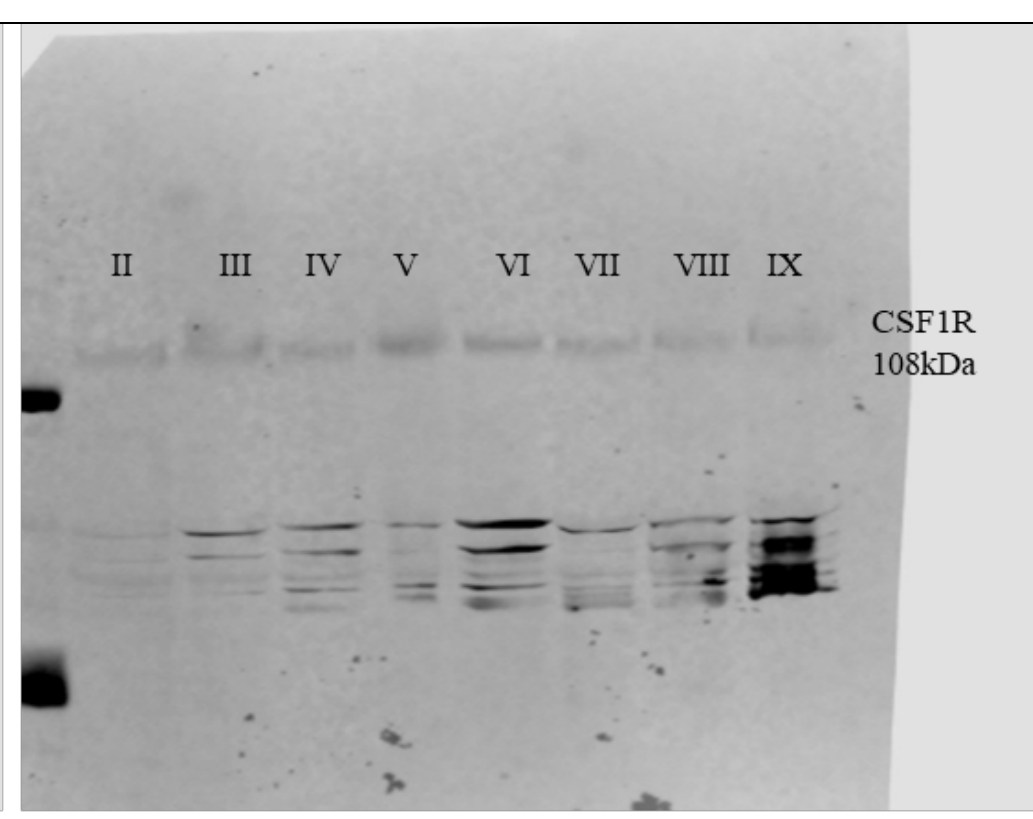

Gel 4		CT		EOAD			LOAD			
Well no	I	II	III	IV	V	VI	VII	VIII	IX	X
Cases	Ladder	GL15	GL2	GL25	GL13	GL1	GL10	GL8	GL6	RIPA
Lysate loaded	07 µL	41.2 µL	41.2 µL	41. µL	41.2 µL	41.2 µL	41.2 µL	41.2 µL	41.2 µL	40 µL
Protein concentration		25ug	25ug	25ug	25ug	25ug	25ug	25ug	25ug	
Primary Antibody	CSF1R (1:700), Anti-CSF-1-R antibody [EPR20634], Monoclonal, ab239079									
Secondary antibody	800CW (1:10000), Goat anti-Rabbit									
Primary Antibody	Beta-Actin (1:1000), anti-Mouse, MAB1501 Millipore Actin									
Secondary antibody	680RD (1:10000), Goat anti-Mouse									

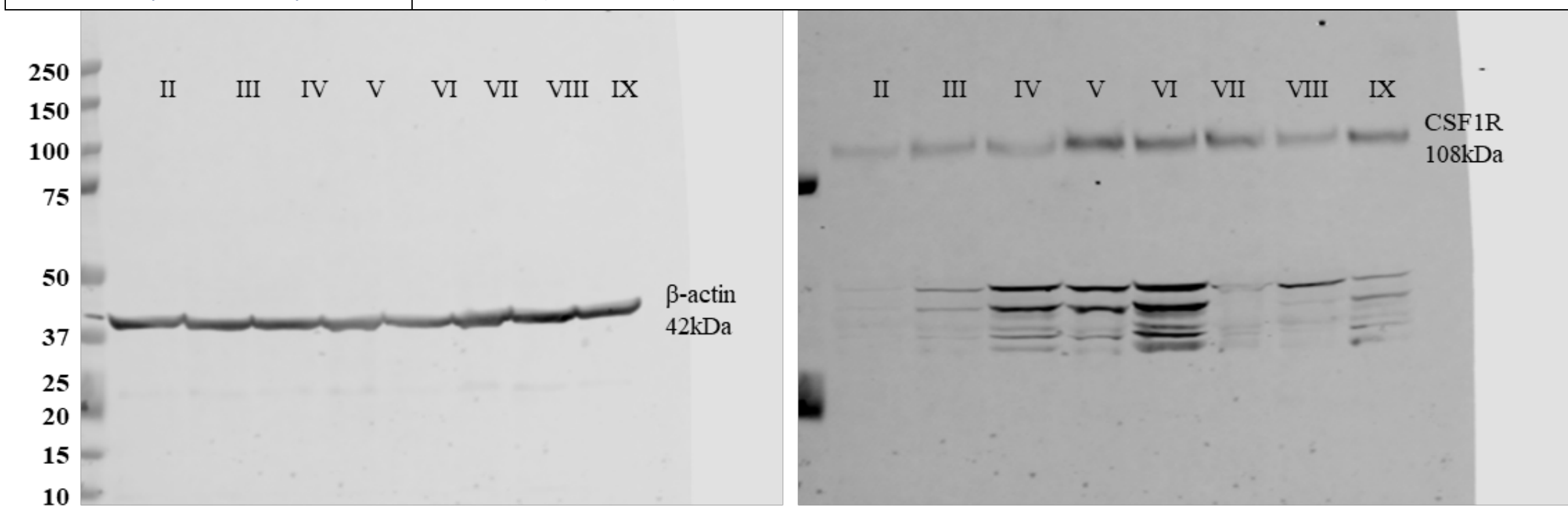

Fig. S2. Quantitative analysis of CSF1R protein expression by Western blot. Immunoblot images and corresponding tables showing CSF1R (108 kDa) and β-actin (42 kDa) in the inferior frontal gyrus (IFG) from four healthy controls (CT), six early-onset Alzheimer's disease (EOAD), and six late-onset Alzheimer's disease (LOAD) cases.
